# Genome-wide by trait interaction analyses with neuroticism reveal chronic pain-associated depression as a distinct genetic subtype

**DOI:** 10.1038/s41398-025-03331-5

**Published:** 2025-03-29

**Authors:** Sandor Krause, Dora Torok, Gyorgy Bagdy, Gabriella Juhasz, Xenia Gonda

**Affiliations:** 1 Department of Pharmacodynamics, Faculty of Pharmaceutical Sciences, Semmelweis University, Budapest, Hungary; 2 Department of Psychiatry and Psychotherapy, Semmelweis University, Budapest, Hungary; 3 Center of Pharmacology and Drug Research & Development, Semmelweis University, Budapest, Hungary; 4 NAP3.0-SE Neuropsychopharmacology Research Group, Hungarian Brain Research Program, Semmelweis University, Budapest, Hungary; 5 Department of Clinical Psychology, Semmelweis University, Budapest, Hungary

**Keywords:** Depression, Genetics

## Abstract

The frequent co-occurrence of chronic pain (CP) and depression is a well-known phenomenon, supported by both the high prevalence of major depression among CP patients and studies describing a substantial genetic correlation between the two phenotypes. Neuroticism, a trait characterised by maladaptive stress responses and a tendency to experience negative emotions, has been linked to both depression and the experience of pain. This study aimed to determine whether depression associated with CP represents a genetically distinct subtype and to explore the role of neuroticism in modulating genetic susceptibility to depression. To address these questions, we performed genome-wide association analyses for current depression utilising the UK Biobank dataset, followed by genome-wide by trait interaction analyses to assess the interaction effect of neuroticism, and polygenic risk score analyses to compare predictions. Our findings suggest that CP-related depression is a valid subtype of depression. In association with current depression, we identified a total of 49 novel genetic risk polymorphisms meeting the genome-wide significance threshold, including variants involved in synaptic plasticity and transcriptional regulation. Additionally, our results support that neuroticism has a prominent role in modulating the genetic risk of current depression independently of CP, which highlights the importance of considering personality traits and stress factors in understanding the genetic background of complex and heterogeneous phenotypes like depression.

## Introduction

The frequent co-occurrence of chronic pain (CP) and depression is a well-known phenomenon. The mean prevalence of major depressive disorder (MDD) among patients with CP is reported to be as high as 50 percent [1, 2]. Prior research has indicated a substantial genetic correlation between CP and depression [3–6]. Furthermore, Johnston et al. [7] have demonstrated a causal effect of multisite CP on MDD with widespread pleiotropy. Other authors emphasised the differentiation between chronic and recent pain [8, 9], which was substantiated by the observation that the polygenic risk score (PRS) for MDD only predicted the latter. Based on these findings, Campos et al. [8] conceptualised a depression subtype characterised by comorbidity of CP and poor treatment response.

Our research group has previously described a transitive relationship between pain-related variables and lifetime depression mediated by current depressive symptoms and, specifically in the case of headache, by neuroticism [10]. The association between neuroticism and the experience of pain is widely recognized [4, 6, 11]: trait neuroticism may predispose to maladaptive coping [12], lower pain thresholds [13], and result in a hypervigilance towards pain [14]. Furthermore, a twin study conducted by researchers at VU Amsterdam suggested that a neurotic personality may increase the risk of developing migraine [15].

Trait neuroticism is a relatively stable and conveniently assessable [16, 17] aspect of an individual’s personality, which affects a wide range of psychological processes beyond pain perception [11]. Most importantly, it contributes to poor adaptation and reaction to stressful conditions and life events in general, and to an increased likelihood of experiencing negative emotions. There is extensive research confirming the association between high neuroticism and the development and recurrence of depression in part related to stress sensitivity and reactivity and difficulties in regulating emotions [18]. Furthermore, longitudinal studies have reported that trait neuroticism predicts both the onset and recurrence of major depressive episodes independently of environmental stress [19]. Subsequent meta-analyses have further confirmed this relationship, also revealing that neuroticism contributes to depression through increased risk of maladaptive coping strategies and rumination [20, 21]. Very recently, the central role of neuroticism in the development of affective disorders has been reconfirmed [22]. Importantly, genetic studies have also pointed toward an association between neuroticism and depression [23].

In our present study, we wanted to investigate if depression occurring in the context of CP is a distinct genetic subtype of depression. In order to test this concept, we compared the genetic architecture of depression in two groups distinguished by the occurrence or absence of CP using genome-wide association analysis (GWAS). Furthermore, we hypothesised that the interaction effect of the genetic variation and neuroticism on depression would be more prominent in the CP group, which we investigated by performing a genome-wide by trait interaction study. Finally, we validated whether PRS scores calculated from the results of the above GWAS studies would be able to predict the presence of current depression and the presence of a depression diagnosis as a function of CP in an independent target sample.

## Materials and methods

### Participants

We used the UK Biobank dataset (Application Number 1602) as a foundation for our association analyses. As our analyses, specifically calculating and testing PRS, required a discovery and a target dataset that were independent of one other at the individual level, we divided the UK Biobank cohort into two samples according to the availability of phenotypic data: the discovery sample comprised those with no missing data on the Patient Health Questionnaire (PHQ-9) assessing current depression severity, while all others with missing data were included in the target sample. Both samples underwent the same quality control steps.

We performed a phenotype-based filtering to exclude possible confounders: we excluded every individual with a medical history of bipolar disorder, any psychotic condition, mental retardation, or substance use disorder using ’First occurrences’ (Category 2405) and UK Biobank data-fields 20002 and 20126 [24]. The data-fields of Category 2405 contain data showing the ‘first occurrence’ of any code mapped to 3-character ICD-10, aggregating primary care data, hospital inpatient data, death register records, and self-reported medical condition codes derived from data-field 20002, reported at the UK Biobank assessment centre visit. Whereas data-field 20126 contains data on bipolar and major depression status, including probable cases, based on prior research at the University of Glasgow [25].

Furthermore, we only included participants with white British ancestry (data-field 22006) who had no missing values regarding sex (data-field 31), age (data-field 21003), genotyping array (data-field 22000), and the phenotypes of interest resulting in a total of 121,574 subjects in the discovery sample, and 196,167 subjects in the target sample.

All participants provided written informed consent, and all procedures were carried out in accordance with the Declaration of Helsinki. Details of the data collection were previously outlined by Bycroft and colleagues [26]. Ethical approval was given by the National Research Ethics Service Committee North West–Haydock. Further information on sample characteristics and the number of subjects excluded can be found in Supplementary Table 1.

### Phenotypes

For grouping, we derived a dichotomous variable (CP status) based on UK Biobank data-fields 3799, 4067, 3404, 3571, 3741, 3414, 3773, and 2956, which measured the different types of pain experienced by participants for more than 3 months [5]. If any of the above items indicated pain, we assigned the subject to the CP group (*N* = 47,313). If all questions were answered in the negative, the subject was sorted into the no-pain (NP) group (*N* = 74,261). As our data on CP do not reflect its temporality relative to the diagnosis of MDD, we have opted to measure genetic susceptibility to current depression rather than lifetime depression. To assess current depression, we used the total score of PHQ-9 (UK Biobank data-fields 20514, 20510, 20517, 20519, 20511, 20507, 20508, 20518, and 20513) as primary phenotype which has been proven to be a reliable and valid measure of depression severity [27] with good internal consistency (Cronbach’s alpha = 0.845). Neuroticism trait was measured by 12 items (data-fields 1920, 1930, 1940, 1950, 1960, 1970, 1980, 1990, 2000, 2010, 2020, and 2030) from the Eysenck Personality Inventory Neuroticism scale (EPIN-R) [16, 25], which also has good internal consistency and a Cronbach’s alpha of 0.834. We classified neuroticism further into ‘low’, ‘medium’, and ‘high’ categories for visualisation purposes, as described in the supplementary materials by Hullam and colleagues [10].

In the target sample, where PHQ-9 data were not available, we used the paper of Hullam et al. [10] as a reference and measured current depression using the ‘current depressive symptoms score’ based on UK Biobank data-fields 2050, 2060, 2070, and 2080. In addition, in the target sample, we used lifetime depression derived from ‘First occurrences’, based on ICD-10 codes F32 (data-field 130894) and F33 (data-field 130896).

To test for multicollinearity, we computed a matrix of Pearson’s correlation and we found a weak but significant correlation between PHQ-9 and EPIN-R scores in both discovery samples with a coefficient of 0.4002 (*p* < 2.2 × 10^−16^, CI: 0.3918–0.4085) in for CP and 0.3801 (*p* < 2.2 × 10^−16^, CI: 0.3733 - 0.3868) for the NP group. In our target sample, we found a similar correlation between EPIN-R and ‘Current depression score’ with a Pearson’s coefficient of 0.5505 (*p* < 2.2 × 10^−16^, CI: 0.5474–0.5535). Since all of these correlation coefficients are small, they do not interfere with the interpretation of the results.

### Genetic data

We followed the same genomic quality control steps for both genetic datasets as described in the supplementary materials of Eszlari and colleagues [28]. We performed filtering steps (minor allele frequency, Hardy-Weinberg equilibrium, missingness, and linkage disequilibrium pruning) and carried out kinship check, sex check, heterozygosity outlier detection, and principal component analysis [28]. Regarding UK Biobank data, 38,939 samples remained in the CP group, counting 6,214,289 genetic variants, and 61,641 samples in the NP group, counting 6,223,429 genetic variants, which were consequently used in our genome-wide association analyses (GWAS).

In the case of the target dataset, we excluded genetic variants on the sex chromosomes and performed filtering based on HapMap3+ variants recommended by Privé and colleagues [29], which provide good coverage of the whole genome. After matching the variants in the summary statistics and the variants in the target sample, 972,321 single-nucleotide polymorphisms (SNP) remained in the CP group and 974,515 SNPs in the NP group, which served as a basis for the polygenic risk calculation.

### Analyses

#### SNP-level analyses

We performed separate GWASs in the CP and NP groups of the discovery sample to examine the direct effect of genetic variants on the PHQ-9 score, followed by a genome-wide by trait interaction analysis to assess the SNP × Neuroticism effect on the same phenotype. In order to eliminate the effect of neuroticism on the SNP main effect, we included it as a covariate in addition to the top ten genomic principal components (PC), sex, age, and genotyping array. All SNP-based association tests were computed using linear regression models in PLINK 2.0 (www.cog-genomics.org/plink/2.0/) [30, 31]. In this way, a total of 4 models were tested and taken into account when adjusting for multiple comparisons.

#### Genetic correlation

We used the SumHer software (https://dougspeed.com/sumher/) [32], assuming LDAK-Thin heritability model [33], to estimate genetic correlation between the two GWAS summary statistics, containing information from the discovery sample about the genetic predisposition to current depression in the NP and CP groups.

#### Functional analyses

We conducted functional annotation for all genetic variants within genomic regions identified by lead SNPs by employing FUMA v1.5.2 (https://fuma.ctglab.nl/) [34, 35]. Lead SNPs were defined as independent SNPs (R^2^ ≤ 0.5) within a locus that had the lowest p-value within a 10,000 base pair range. A significance threshold of *p* ≤ 5.0 × 10^−8^ was applied for lead SNPs, while *p* ≤ 1.0 × 10^−5^ was considered for suggestive significance. To adjust for multiple comparisons, we used a Bonferroni-corrected threshold of *p* ≤ 2.0115 × 10^−9^ for the CP group and *p* ≤ 2.0085 × 10^−9^ for the NP group.

#### Gene-based tests

By performing gene-based tests with MAGMA v1.08 (https://ctg.cncr.nl/software/magma) [36], SNP-based p-values were aggregated into gene-based p-values using a SNP-wise mean model. SNPs were assigned to protein-coding genes based on their positions, with gene boundaries extended by 10,000 base pairs. The number of mapped genes was 19,884 in the CP group and 19,886 in the NP group, resulting in Bonferroni-adjusted thresholds of *p* ≤ 6.2865 × 10^−7^ and *p* ≤ 6.2858 × 10^−7^, respectively.

#### Gene-set analyses

The MAGMA gene-set analysis [36] tests whether the genes in a gene-set are jointly associated with the phenotypes by regressing transformed gene p-values on the gene-set indicator variable. Gene-set definitions were derived from MsigDB v2023.1.Hs [37], including curated gene-sets (C2) and Gene Ontology gene-sets [38, 39] (C5 GO:BP - biological process; C5 GO:CC - cellular component; C5 GO:MF - molecular function). In our analyses, this entailed 17,012 gene-sets and thus a Bonferroni-corrected p-value threshold of 7.3478 × 10^−7^ for both groups.

To overcome the limitations of using only position-based assignments and protein-coding genes within MAGMA, top SNPs of SNP-level tests were mapped to genes based not only on position but also on functional annotations. The mapped genes were further analysed using hypergeometric tests to check for overrepresentation within predefined gene-sets. In these enrichment analyses conducted through the ’GENE2FUNC’ function of FUMA [34, 35], a Benjamini–Hochberg FDR adjusted threshold of *p* ≤ 0.05 was set for each subcategory of gene-sets. For this study, the following MsigDB subcategories were considered: C3 MIR (microRNA targets), C3 TFT (transcription factor targets), and the above-mentioned three subcategories from Gene Ontology gene-sets.

#### Calculation of polygenic risk scores

We used the automatic model of the LDpred2 software [29, 40] (LDpred2-auto in short), which calculates polygenic risk by inferring and aggregating the posterior mean effect size of each SNP, based on prior knowledge from the above GWAS summary statistics, taking into account information on linkage disequilibrium (LD) and genetic architecture. This method requires two hyper-parameters: heritability and the proportion of causal variants, which LDpred2-auto can infer directly using multiple iterations and an initial heritability estimate generated by LD Score regression [41].

We calculated PRS scores on the target sample providing effect sizes from our own GWAS summary statistics on PHQ-9 total score. As both the target sample and the GWAS summary statistics were sourced from the UK Biobank database, we only performed a basic SNP-based harmonisation restricted to HapMap3+ variants [29]. We carried out a genome-wide computation running 50 Gibbs sampler chains in parallel. As a result, 2 separate PRS scores were obtained, namely PRS_CP_ and PRS_NP_, which encompassed the genetic predisposition to current depression in the CP and NP groups, respectively.

#### Evaluation of polygenic risk scores

We tested each PRS score with generalised linear models in the target sample, using the PRS score and the same set of covariates as predictors as in the GWAS analyses regarding SNP main effects. We then computed an estimate of the predictive ability of the PRS score by subtracting R^2^ between this model and the corresponding covariate-only model and denoted it ΔR^2^. In a subsequent step, we performed another set of regression analyses to assess the explained phenotypic variance by the interaction of neuroticism and the genetic risk for depression, embodied in our PRS scores. Eqs 1, 2 provide the R formulas of the main and interaction models, respectively.

**Main model:**

1 $${Outcome} \sim {Age}+{Sex}+{Array}+{PCs}+{Neuroticism}+{PRS}$$

**Interaction model:**

2 $${Outcome} \sim {Age}+{Sex}+{Array}+{PCs}+{Neuroticism}+{PRS}+{PRS:Neuroticism}$$

For our PRS analyses in the target sample, two outcome variables were considered: current depression as measured by the current depressive symptoms score, and lifetime depression reflecting the presence of a depression diagnosis. We used linear regression for the first and logistic regression for the second variable. In order to allow for a comparison of the goodness of fit of linear and logistic regression models, a pseudo R^2^ was calculated for the latter using the method proposed by Nagelkerke [42]. Finally, we fitted additional models to compare the predictive performance of PRS scores across subsamples stratified by CP status (CP target subsample: *N* = 85,420; NP target subsample: *N* = 110,747). Altogether, a total of 24 generalised linear models were applied, and p-values were adjusted accordingly using Bonferroni correction.

#### Data cleaning, statistics and visualisation

Data cleaning, descriptive statistics, and regression analyses testing the predictive effect of the calculated PRS scores were conducted using R version 4.1.2 (https://www.r-project.org/) [43] and the tidyverse meta-package [44]. The R packages bigstatsr (v1.5.12) and bigsnpr (v1.12.2) were used as part of the LDpred2 method [45]. Manhattan plots were created via topr (v2.0.2) [46, 47], all other plots were made using ggplot2 [48]. Nagelkerke’s R^2^ was computed using the R package fmsb (v0.7.6) [49, 50].

## Results

### Main effect analyses in the NP and CP groups

#### SNP-level findings

**Fig. 1:**
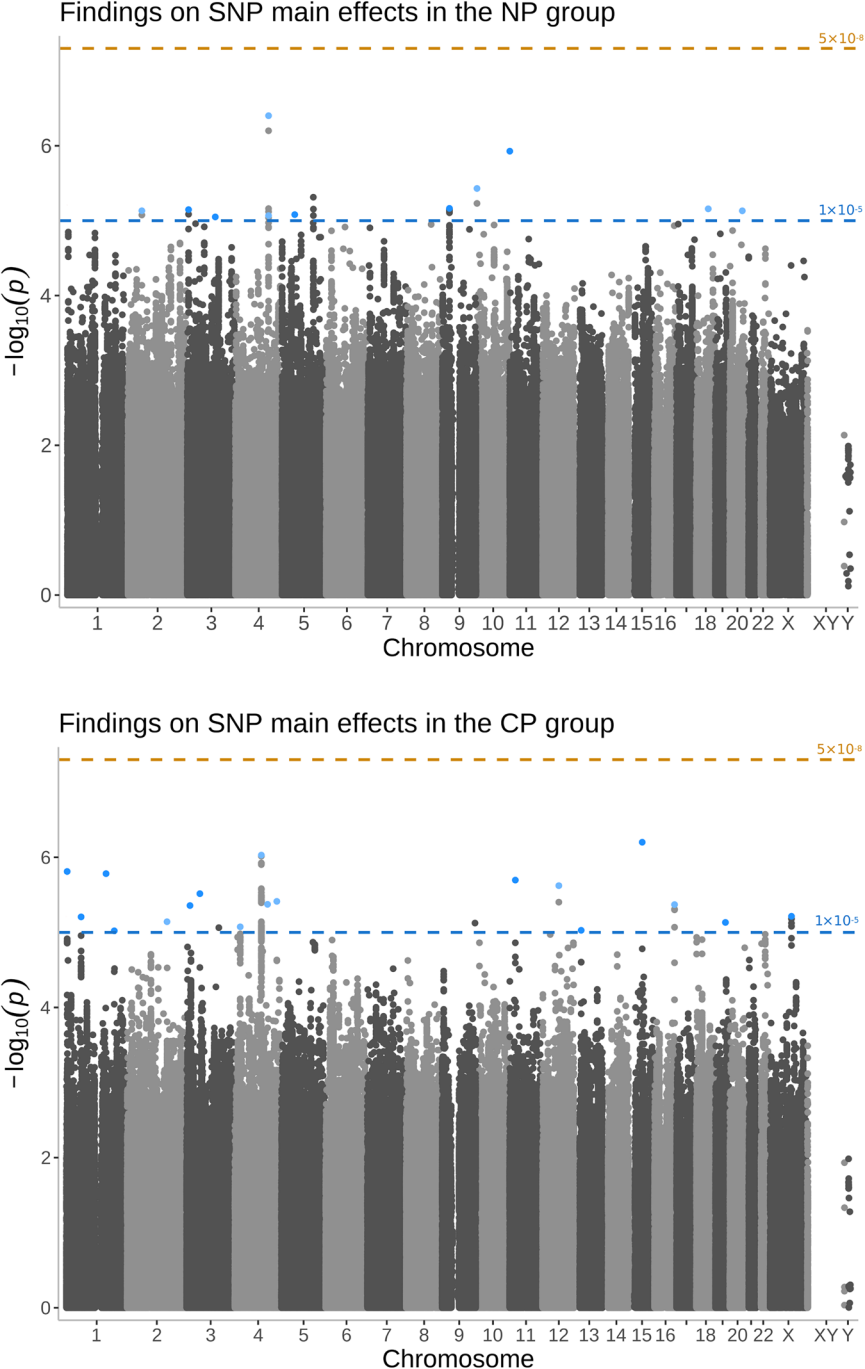
Manhattan plots presenting genetic variants with suggestive significance identified by our main effect analyses in the no-pain (NP) and chronic pain (CP) groups. The plot above displays results from the no-pain (NP) group, while the plot below displays data from the chronic pain (CP) group. The x-axis shows the physical position of genetic variants, arranged by chromosomes. The y-axis shows the level of significance on a negative logarithmic scale, with a blue line denoting the suggestive significance threshold (*p* ≤ 1.0 × 10^−5^) and an orange line denoting the genome-wide significance threshold (*p* ≤ 5.0 × 10^−8^). The highlighted single-nucleotide polymorphisms (SNP) are lead SNPs, each within a locus of 10,000 base pair range, colour-coded according to significance.

Regarding the main effect of SNPs, 18 variants in the NP group and 11 in the CP group reached suggestive significance with no overlap between them. None of these variants met the genome-wide significance threshold or survived multiple testing correction. Fig. 1 presents these findings in a Manhattan plot. A complete list of the genetic variants organised by groups can be found in Supplementary Table 2 and Supplementary Table 3.

#### Results of MAGMA analyses

Gene-based tests and gene-set analyses performed using MAGMA yielded no significant results in either group.

#### Gene-set enrichment analyses

In the NP group, a single gene-set from GO:BP was identified (Supplementary Table 6), whereas in the CP group 5 gene-sets from GO:CC and 10 gene-sets from GO:MF remained significant (Supplementary Table 7).

### Genetic correlation for current depression between the CP and NP groups

We found a genetic correlation of 0.9102 (SE = 0.2048) between the two GWAS summary statistics, with a heritability estimate of 0.0352 (SE = 0.0096) for current depression in the NP group and 0.0767 (SE = 0.0148) in the CP group.

### Interaction analyses between genetic variants and neuroticism in the NP and CP groups

#### SNP-level findings

**Fig. 2:**
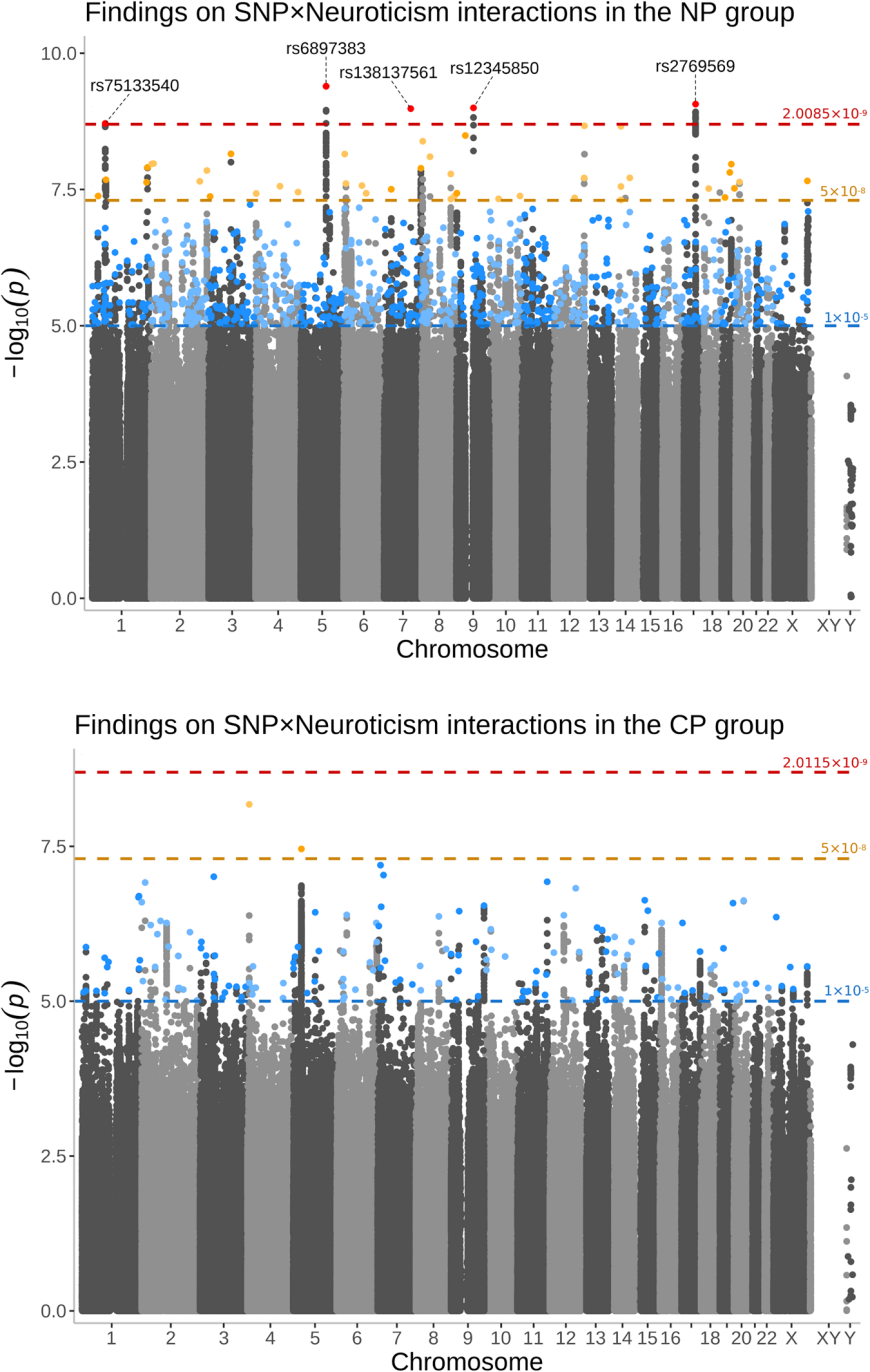
Manhattan plots presenting significant genetic variants identified by our interaction analyses between genetic variants and neuroticism in the no-pain (NP) and chronic pain (CP) groups. The plot above displays results from the no-pain (NP) group, while the plot below displays data from the chronic pain (CP) group. The x-axis shows the physical position of genetic variants, arranged by chromosomes. The y-axis shows the level of significance on a negative logarithmic scale, with a blue line denoting the suggestive significance threshold (*p* ≤ 1.0 × 10^−5^), an orange line denoting the genome-wide significance threshold (*p* ≤ 5.0 × 10^−8^), and a red line denoting the Bonferroni-corrected threshold (*p* ≤ 2.0115 × 10^−9^ for the CP and *p* ≤ 2.0085 × 10^−9^ for the NP group, respectively). The highlighted single-nucleotide polymorphisms (SNP) are lead SNPs, each within a locus of 10,000 base pair range, colour-coded according to significance. Lead SNPs surviving multiple testing correction were annotated with the corresponding reference SNP identifier (rsID) assigned by dbSNP.

Regarding SNP × Neuroticism interactions, 165 variants reached suggestive significance in the CP group, of which 2 variants (rs61096498: beta = −0.0940, *p* = 6.6414 × 10^−9^; rs10941193: beta = 0.1602, *p* = 3.4766 × 10^−8^) met the genome-wide significance threshold, but did not survive the Bonferroni-correction (Supplementary Table 2). In the NP group, of the 777 suggestive significant variants, 47 reached the genome-wide significance threshold and 5 (rs6897383: beta = −0.0346, *p* = 4.0404 × 10^−10^; rs2769569: beta = 0.0321, *p* = 8.5806 × 10^−10^; rs12345850: beta = 0.0799, *p* = 1.0054 × 10^−9^; rs138137561: beta = 0.1051, *p* = 1.0401 × 10^−9^; rs75133540: beta = −0.0908, *p* = 1.9418 × 10^−9^) remained significant after adjusting for multiple testing (Supplementary Table 3). Furthermore, we identified only 1 overlapping variant between the interaction analysis and the main effect analysis results in each group, namely rs35519714 in the CP group and rs72762916 in the NP group. A Manhattan plot summarising the SNP-level findings of these interaction analyses is presented in Fig. 2.

#### Results of MAGMA analyses

Gene-based tests showed significant associations with 16 genes in the NP group, and with 1 gene (*SDC2*: *p* = 3.078 × 10^−7^) in the CP group (Supplementary Table 4). Among the former, the top four significant findings were *ANXA13* (*p* = 5.0656 × 10^−9^), *SRSF10* (*p* = 1.4593 × 10^−8^), *TSHZ2* (*p* = 2.2068 × 10^−8^), and *HDAC4* (*p* = 2.4605 × 10^−8^). MAGMA gene-set analyses identified a single significant gene-set (GOBP_HISTONE_H3_K9_DIMETHYLATION: *p* = 2,18 × 10^−6^) in the CP group, compared to none in the NP group (Supplementary Table 5).

#### Gene-set enrichment analyses

In the NP group, we identified 271 microRNA targets and 25 transcription factor targets, as well as 5 GO gene-sets: 2 from GO:BP, 2 from GO:CC, and 1 from GO:MF, respectively. In comparison, the results for the CP group showed 18 significant gene-sets from GO:BP, 7 from GO:CC, and 2 from GO:MF, with 4 additional transcription factor targets. A complete list of identified gene-sets is presented in Supplementary Table 6 and Supplementary Table 7, organised by groups.

### Polygenic risk score analyses in the target sample

#### Predictive effect of PRS scores on current depression

**Fig. 3:**
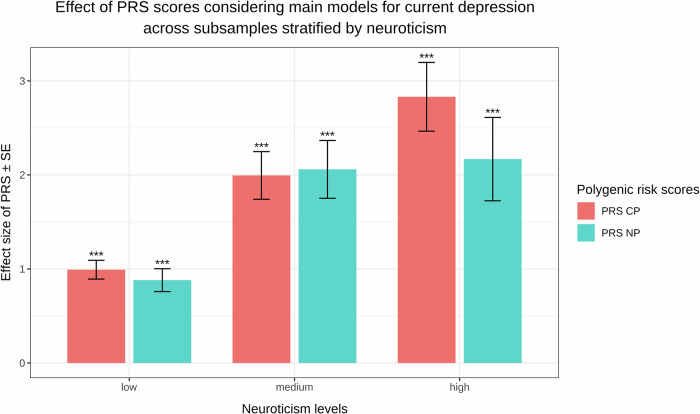
A clustered bar chart illustrating the predictive effect of polygenic risk scores (PRS) representing an aggregated genetic risk for depression based on data from the no-pain (PRS_NP_) and chronic pain (PRS_CP_) groups on current depression in the total target sample stratified by neuroticism. The x-axis ranks the categories from low neuroticism to high neuroticism. The y-axis shows the effect sizes of PRS scores derived from our main regression models, with an error bar indicating standard error (SE). Following conventions, asterisks on top of the bars indicate the level of significance (*p ≤ 0.05, **p ≤ 0.01, ***p ≤ 0.001). The colours specified in the legend represent results for PRS scores calculated in the chronic pain (CP) and no-pain (NP) groups, respectively.

Both PRS_NP_ and PRS_CP_, representing an aggregated genetic risk for depression based on data from the NP and CP groups respectively, made a significant contribution to the CDS score of the total target sample (PRS_NP_: beta = 1.4197, p_adj_ < 2 × 10^−16^, ΔR^2^ = 4 × 10^−4^; PRS_CP_: beta = 1.5671, p_adj_ < 2 × 10^−16^, ΔR^2^ = 6.538 × 10^−4^). When looking at the results of the interaction models, the main effects of these PRS scores seemingly diminished and were taken over by the interaction effect of PRS × Neuroticism (PRS_NP_ × Neuroticism: beta = 0.2569, p_adj_ = 5.7953 × 10^−9^, ΔR^2^ = 1 × 10^−4^; PRS_CP_ × Neuroticism: beta = 0.2794, p_adj_ = 1.3019 × 10^−15^, ΔR^2^ = 3 × 10^−4^). These findings were independent of CP status and remained consistent across subsamples. The predictive effect of PRS scores on current depression is visualised in Fig. 3, stratified by neuroticism levels to better illustrate their interaction. A comprehensive presentation of these results is available in Supplementary Table 8.

#### Predictive effect of PRS scores on lifetime depression

**Fig. 4:**
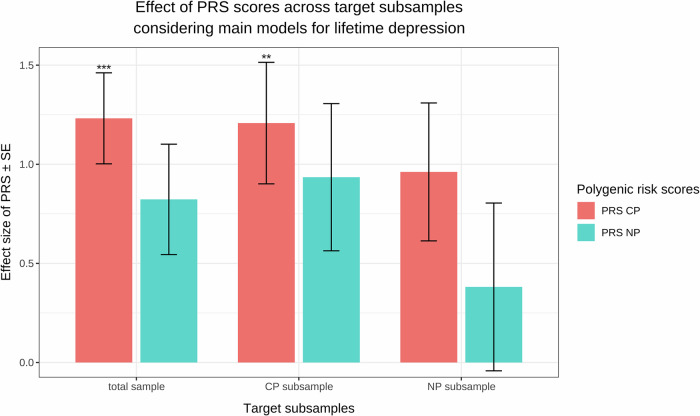
A clustered bar chart illustrating the predictive effect of polygenic risk scores (PRS) representing an aggregated genetic risk for depression based on data from the no-pain (PRS_NP_) and chronic pain (PRS_CP_) groups on lifetime depression in the target sample stratified by chronic pain status. The x-axis lists the total target sample and two target subsamples based on the presence and absence of chronic pain. The y-axis shows the effect sizes of PRS scores derived from our main regression models, with an error bar indicating standard error (SE). Following conventions, asterisks on top of the bars indicate the level of significance (*p ≤ 0.05, **p ≤ 0.01, ***p ≤ 0.001). The colours specified in the legend represent results for PRS scores calculated in the chronic pain (CP) and no-pain (NP) groups, respectively.

Regarding our logistic regression analyses, we observed that PRS_CP_ had a significant predictive effect in the main model (beta = 1.2317, p_adj_ = 1.9192 × 10^−6^, ΔR^2^ = 3 × 10^−4^), while PRS_NP_ did not (beta = 0.8228, p_adj_ = 7.5235 × 10^−2^, ΔR^2^ = 9.09 × 10^−5^). When considering target subsamples stratified by CP status, the main effect of PRS_CP_ appeared to be significant only in the CP target subsample (CP target subsample: beta = 1.2077, p_adj_ = 1.9597 × 10^−3^, ΔR^2^ = 3.2 × 10^−4^; NP target subsample: beta = 0.9614, p_adj_ = 1.3734 × 10^−1^, ΔR^2^ = 1.686 × 10^−4^). In contrast, PRS_NP_ did not contribute to the explained phenotypic variance in either subsample. Looking at the interaction models, we found no significant effect on lifetime depression for either PRS_NP_ or PRS_CP_ in interaction with neuroticism. The predictive effect of PRS scores on lifetime depression is illustrated in Fig. 4. For more details, see Supplementary Table 9.

## Discussion

In our study, we investigated the genetic background of depression occurring in the presence or absence of chronic pain, and how trait neuroticism interacts with genetic variation on current depression in these two depression subtypes. Finally, we also examined if PRS scores calculated in the above analyses significantly predicted current depression or depression diagnoses in an independent target sample, either independently or in interaction with neuroticism.

### Investigation of the genetic architecture of depression in interaction with neuroticism may suggest distinct subtypes in the presence and absence of chronic pain

In the CP group, we observed higher means for depression and neuroticism scores, and the SNP heritability estimate of current depression was double in the CP group, which can be attributed to the fact that the CP group is more homogeneous, and depression scores, which tend to change dynamically in general, may be more persistent here, representing a continuous depressive state as consequence to CP. Although we found a high genetic correlation between the two groups, this was not unexpected given that we were making a comparison based on the same trait. In comparison, Meng et al. reported genetic correlations across eight pain phenotypes, with estimates ranging from 0.36 to 0.83 [6].

In our main effect analyses we could not identify significant variants, genes, or gene-sets associated with current depression either in the CP or NP groups after correcting for multiple testing.

However, when the interaction effect of neuroticism was considered, a large number of significant variants were identified, especially in the NP group, which, besides confirming the importance of neuroticism in the manifestation of genetic vulnerability, contradicts our hypothesis that neuroticism plays a greater role in the appearance of depressive symptoms in the CP group. In the interaction analyses, regarding the lead SNPs of the risk loci, we did not find any overlapping variants between the CP and NP groups, which once again highlights differences between depression that manifests in the presence and absence of CP.

Of the lead SNPs identified, only one variant has so far been reported in the context of depression, namely rs17634917 in the CP group regarding SNP × Neuroticism interaction, which appears to mediate a predisposing effect. It has been mapped to the *LOC105372465*, *RFPL4A* and *NLRP9* genes, the first two of which regulate transcription and the latter plays a role in the innate immune system. All other hits can be considered as new associations.

### PRS analyses in an independent target sample also support differences between depression in the presence and absence of chronic pain

In addition to the above genome-wide by trait interaction studies suggesting that CP-associated depression may be a distinct depression subtype, our PRS studies pointed in a similar direction. Whereas for current depression, we did not find differences in the predictive power of the two PRS scores, we discovered a meaningful difference in the prediction of lifetime depression: PRS_CP_ predicted depression diagnoses in the CP target subsample, contrary to the NP group, supporting the concept of a distinct depression subtype.

### The role of neuroticism in the manifestation of depressive symptoms is independent of chronic pain status

In line with previous knowledge, our results show that neuroticism has a great influence on the genetic risk of developing depressive symptoms, however, contrary to our expectations, this effect was largely independent of CP status. This former finding is also reflected in the effect of PRS scores on current depression, which is best seen in our interaction models, where the previously significant effect of PRS scores is mediated entirely through the PRS × Neuroticism effect.

### Our analyses confirm variants previously associated with depression

For the main effect analysis in the NP group, we detected one suggestively significant genetic variant mapped to the *DCC* gene. DCC is the receptor of Netrin-1, together they play an important role in guiding the growth cones during adolescent neurodevelopment. There is converging evidence that this signalling pathway may be affected in multiple mental disorders, including MDD [51].

Furthermore, rs72762916, the sole overlapping variant identified with a suggestive significance in the NP group in both the main effect and interaction analysis results, was mapped to *ADARB2*. The *ADARB2* gene encodes an RNA-editing enzyme expressed primarily in inhibitory neurons. Interestingly, in addition to its polymorphisms that predispose to depression [52, 53], it has recently been suggested as a potential contributor to protection from headache [54].

In the CP group, we found two suggestive significant polymorphisms mapped to the genes *ERC2* and *CDH13* respectively, the former playing a role in circadian regulation and neuroplasticity [55], the latter encoding a protein localised to the cell surface that protects against oxidative stress and regulates axon growth during neural development [56]. Both genes have been linked to depression [52, 57, 58], but a polymorphism of the *CDH13* gene has also been associated with an increased risk of migraine [59].

### Several identified variants and genes are in line with previous results concerning the interaction of stress in the background of depression

From our results in the CP group, we highlight two genes: *SORCS2* and *SDC2*. In interaction with neuroticism, we identified two protective polymorphisms mapped to the former gene: rs4689765 with a suggestive significance and rs61096498, which was significant at the genome-wide level. *SORCS2* is known to play a role in the BDNF-dependent synaptic plasticity. It has been already linked to neuroticism [60], but more interestingly, Chen et al. [61] found that the hippocampal overexpression of *SORCS2* repressed chronic stress-induced depressive-like behaviours in mice. Our observations suggest that *SORCS2* might be affected in CP-induced depression as well.

*SDC2*, on the other hand, is a member of the syndecan family, involved in cell binding, cell signalling, and cytoskeletal organisation, which we identified in association with current depression via MAGMA gene-based tests, drawing on our results from the genome-wide by trait interaction analysis. It has been reported in the context of inflammation and blood-brain barrier disruption [62] and as a candidate gene for post-traumatic stress disorder (PTSD) [63] and suicidal ideation [64]. It is well documented that CP has a higher comorbidity rate with PTSD [65], moreover, prior research has demonstrated that targeting symptoms of PTSD may improve pain [66], implying common neurobiological mechanisms between the two phenotypes.

Among the results of the interaction analysis in the NP group, we identified three significant variants mapped to the *LOC102724945* RNA gene and two variants, one of which was only suggestive, mapped to the *TRPM3* gene. *TRPM3* encodes a cation-selective channel and is involved in cellular calcium signalling and homeostasis. Coleman et al. [67] reported polymorphisms in the aforementioned genes which conveyed a susceptibility to MDD only in the subgroup that had not experienced trauma. Our results both originated from the NP group that had previously been pruned of CP, and the same genetic variants had no significant effect in the CP group. This again suggests, albeit indirectly, a link between the neurobiology underlying trauma and CP.

### Our analyses identify genes with pleiotropic effects and underscore the importance of transcriptional regulation in the development of depression

We mapped 3 genetic variants among the top findings of the main effect analysis in the NP group to the *CSMD1*, *HDAC4* and *RGS6* genes, respectively. *CSMD1* is an integral membrane component affecting several processes, including tumour suppression, complement-mediated synaptic pruning, and immune-related signalling [68]. *RGS6* regulates G-protein signalling and is enriched in hippocampal and cortical neurons. It was also implicated as a regulator of 5-HT_1A_R signalling, thus modulating anxiety and depression [69]. Lastly, *HDAC4* is a transcriptional regulator which is also involved in neuronal synaptic plasticity and memory formation [70].

What these genes have in common is that they have been all reported in the context of pleiotropy, affecting multiple mental disorders [71, 72]. In contrast, our CP group is a more homogenous and specially selected sample, where SNPs mediating general susceptibility may play a minor role.

*HDAC4* was also reported by the MAGMA gene-based tests in the NP group, where 5 out of 16 genes were involved in transcriptional regulation (*SRSF10*, *TSHZ2*, *HDAC4*, *EXOSC5*, *PNRC2*). Their importance is reflected in the abundance of microRNA and transcription factor targets in the gene-set enrichment analysis results, which were not replicated in the CP group.

### Potential importance of findings for clinical practice and future application

Given that depression is a highly heterogeneous disorder with a multifactorial background, where different symptomatic manifestations are underlain by highly divergent neurobiological processes and disturbances and are subject to the interacting effects of distal and proximal stressors, both a deeper understanding of its potential subtypes and the identification of biomarkers or fingerprints are urgently warranted. Furthermore, depression associated with somatic disorders or comorbidities is considered a clinically distinct entity. Such somatic comorbidities may have a bidirectional relationship with depression, may present as a chronic stressor increasing risk, and may complicate the treatment of both conditions. Therefore, understanding the relationship between CP and depression, as well as the role of personality in this relationship, may bring us closer to characterizing subtypes, providing important insight into risk prediction, biomarker signatures for screening, and potentially novel treatment targets. Based on the accumulating evidence supported by our findings, CP-associated depression should also be considered a distinct clinical entity in clinical studies on depression treatment. As the next steps, responses to existing treatments in distinct subgroups of depression, including the one associated with CP, should be separately investigated, paving the way towards a much-needed precision approach in psychiatry.

### Limitations

Our results must be viewed and interpreted in the context of a number of limitations which may also influence the generalizability of our findings.

First, there is evidence that there are genetic differences between CP subtypes [6, 73], however, we chose not to take these differences into account in order to include as many participants as possible in our analyses, increasing statistical power. Future studies should take these questions into consideration.

Second, our analyses were performed using the UK Biobank database. Although the UK Biobank is one of the most extensive available population health resource databases, it is also subject to several biases that must be kept in mind when generalizing our findings. The UK Biobank cohort is ethnically predominantly White European (approx. 94%), aged during initial recruitment 40–69 years, participants are healthier, wealthier, and better educated compared to the general population. In addition to the above limitations related to representativeness, there may also be significant selection bias related to recruitment methods and low enrollment rates in the originally invited participants. Finally, the majority of UK Biobank data are self-reported and cross-sectional, including our CP-related variables and both measures for current depressive symptoms. The above-mentioned limitations of the sample should be carefully considered when interpreting our findings and generalizing them to larger populations [74–77].

Third, in addition to the above potential biases related to the characteristics of the UK Biobank sample, we excluded individuals with several mental disorders, including bipolar and psychotic disorders, as well as those with mental retardation and substance use disorders, which should also be considered when applying our findings in the general population. Further studies addressing subject diversity and generalizability are required [78].

## Conclusion

In summary, our findings support that the proposed depression subtype associated with CP has distinct genetic characteristics. Furthermore, our results demonstrated that neuroticism has a prominent role in modulating the genetic risk of current depression independently of CP status, which is less pronounced when considering more robust phenotypes such as lifetime depression. We identified novel genetic risk polymorphisms in association with current depression, including multiple variants involved in synaptic plasticity and transcriptional regulation. Our findings emphasise that the genetic background of complex and heterogeneous phenotypes such as depression is challenging to unravel without considering the context of personality traits and stress factors, given their intricate interrelationships. A deeper comprehension of their interactions and the genetic architecture of depression subtypes may facilitate the development of innovative therapeutic approaches and lead us towards personalised medicine. Further research is needed to replicate our findings within population samples of different ancestries and to investigate the potential differences across CP subtypes.

## Supplementary information

Supplementary tables

## Data Availability

UKB data is available for further research upon application to the data owners: UK Biobank, application number 1602.
